# Field dataset of punctual observations of soil properties and vegetation types distributed along soil moisture gradients in France

**DOI:** 10.1016/j.dib.2022.108632

**Published:** 2022-09-23

**Authors:** Guillaume Gayet, François Botcazou, Jean-Manuel Gibeault-Rousseau, Laurence Hubert-Moy, Sébastien Rapinel, Blandine Lemercier

**Affiliations:** a Unité d'appui et de Recherche Patrimoine Naturel, Office Français de la Biodiversité, Centre National de la Recherche Scientifique, Muséum national d'Histoire Naturelle, 36 rue Geoffroy Saint Hilaire, CP 41, Paris 75 005, France; b Institut Agro Rennes-Angers, INRAE, SAS, 65 rue de Saint-Brieuc, CS84215, Rennes Cedex 35042, France; c LETG, UMR 6554 CNRS, Université Rennes 2, Place du recteur Henri Le Moal, Rennes Cedex 35043, France

**Keywords:** Biodiversity, Natural habitat, Soil, Redoximorphic features, EUNIS, Wetlands

## Abstract

The interface between wetlands and uplands is characterized by gradients in hydrological, soil and biological components. Consequently, the exact spatial distribution of this transitional area is not well known because it often occurs as a fuzzy moisture gradient. However, ecological assessment and conservation require mapping and characterizing this interface to better understand and model biotic and abiotic interactions between wetlands and uplands. To this end, in 2021 and 2022, we observed soil properties and vegetation types along soil moisture gradients throughout the Atlantic, Continental, Mediterranean and Alpine biogeographic regions of France. The dataset contains 2 236 georeferenced plots (accuracy ± 5 m) distributed along 1 088 transects placed along the slope at 377 sites. Each plot in the database is characterized by 21 fields that describe the vegetation habitat type based on the European Nature Information System (EUNIS) and soil properties (i.e. depth of appearance and thickness of redoximorphic features in the soil profile, moisture). These data are useful for researchers and engineers in a variety of disciplines (e.g. Earth and life sciences) to calibrate and validate models to predict the spatial distribution of habitats or to analyze flows.

**Specifications Table**

**Table:** 

Subject	Soil Science, Ecology, Environmental Engineering, Nature and Landscape Conservation
Specific subject area	Nature and Landscape
Type of data	Geolocalized point data
How the data were acquired	The data were collected in the field and characterized by observation of plant species and hand-auger drilling of soils. The geographical coordinates of each plot were recorded using a GPS with a horizontal precision of ± 5 m.
Data format	Raw
Description of data collection	2 236 plots were sampled in 2021 and 2022 along 1 088 transects at the interface between wetlands and uplands across mainland France and Corsica. For each plot, the vegetation habitat type was classified according to EUNIS based on the observed plant species and local environmental conditions. Soil properties include the depth of redoximorphic features in the soil profile, their thickness observed in the soil core down to a depth of 120 cm and moisture observed in the soil core down to a depth of 120 cm.
Data source location	• City/Town/Region: all over biogeographical regions of France [1]
	• Country: mainland France
Data accessibility	Name of the repository: Dryad
	Title of the dataset: dataset of punctual observations along soil moisture gradients in France
	Direct (URL) link to the dataset: https://datadryad.org/stash/dataset/doi:10.5061/dryad.gb5mkkwsd
	Data identification number associated with this dataset: doi:10.5061/dryad.gb5mkkwsd.
Related research article	No related article has been published to date

## Value of the Data

• These data are useful to describe soil moisture gradients at a fine spatial scale in a wide variety of geomorphological contexts.
• The dataset can be used to calibrate and validate spatial distribution or flow-analysis models.
• The dataset can benefit researchers and engineers involved in Earth and life sciences.

## Data Description

**Fig. 1:**
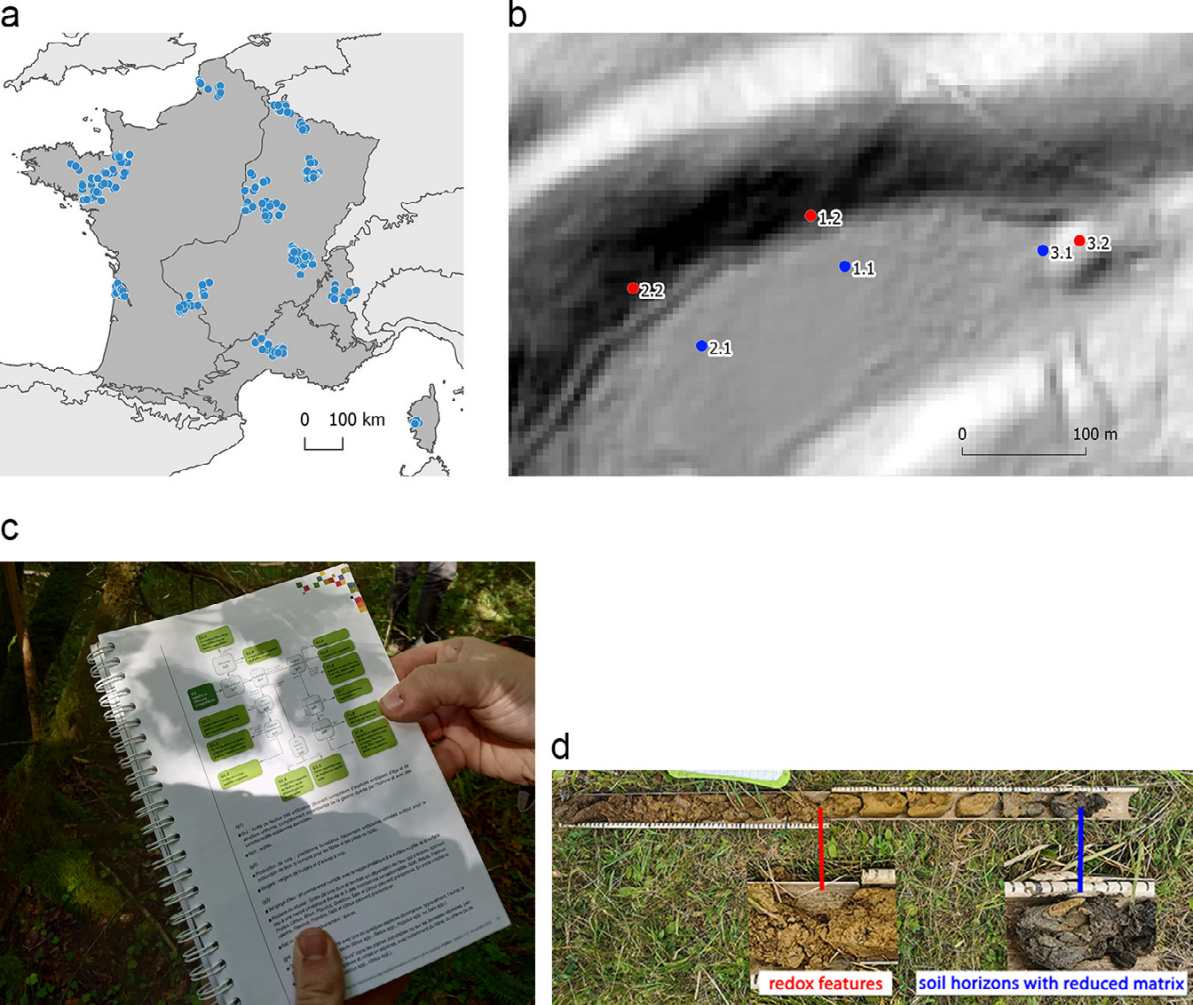
Distribution of survey sites (points) in the four biogeographic regions of France (a) and overview of survey plots at one site (b, blue points: wetlands, red points: non-wetlands). A handbook was used to identify the habitat [3] (c) and soil was described after sampling it (d) in each survey plot.

The dataset is a table in CSV format that contains 21 attribute fields related to plot identification, geographical coordinates, vegetation type and soil properties (Table 1). The spatial distribution of the data and photographs that illustrate the surveys are shown in Fig. 1. In detail, 794 plots were sampled in the Atlantic biogeographical region, 1 052 in the Continental region, 211 in the Alpine region and 179 in the Mediterranean region of France. Fig. 1b represents three transects at one site. Transect 1 was 50 m long and had a difference in elevation of 3 m. It was located in the Continental biogeographical region: the lowest plot (ID 1.1) contained moist or wet oligotrophic grassland (EUNIS code E3.5) with a Histosol soil [2] with peat horizons 0–0.8 m deep, while the highest plot (ID 1.2) contained highly artificial conifer plantations (EUNIS code G3.F) and well-drained soil. Fig. 1d highlights an auger soil profile with distinct redoximorphic mottles 40 cm deep (redox features in red from 40 to 95 cm, soil horizons with reduced matrix in gray from 95 to 120 cm).

**Table 1:** Name and description of the 21 attribute fields in the dataset. The notation ‘ntr’ and ‘na’ in the dataset means ‘nothing to report’ and ‘not available’, respectively.

Field name	Description
id	unique identifier for each field observation.
site	unique site name.
transect_num	transect number at a given site.
plot_num	plot number along a given transect.
X	longitude coordinates in degrees (WGS 84 coordinate system, EPSG 4326).
Y	latitude coordinates in degrees (WGS 84 coordinate system, EPSG 4326).
GPS_acc	accuracy of geographical coordinates (in m). ‘dep’ indicates that the plot was georeferenced using aerial photographs due to field-accessibility issues (e.g. ledge, swamp).
HGM	hydrogeomorphic type from Brinson (1993), expressed in five categories: ‘riverine or estuarine fringe’, ‘depressional’, ‘slope’, ‘lacustrine fringe’, ‘flat’. Brinson, M., 1993. A Hydrogeomorphic Classification for Wetlands (Wetlands Research Program Technical Report No. WRP-DE-4). US Army Corps of Engineers.
habitat_code	Code of habitat type according to the EUNIS classification system (Davies et al. 2004). ‘INV’ indicates that vegetation entirely consists of invasive plant species. Davies, C.E., Moss, D., Hill, M.O., 2004. EUNIS habitat classification revised 2004. Rep. Eur. Environ. Agency-Eur. Top. Cent. Nat. Prot. Biodivers. 127–143.
habitat_name	Name of habitat type according to the EUNIS classification system.
habitat_min_radius	minimum radius of the vegetation patch (in m).
soil_depth	depth of the soil profile (in cm).
soil_redox_depth	depth of occurrence of redox features that covered 5% or more of the soil aggregates (in cm).
soil_redox_thickness	thickness of redox features that covered 5% or more of the soil aggregates (in cm).
soil_reduced_depth	depth of the occurrence of soil horizons with a reduced matrix that covered 95% or more of the soil aggregates (in cm).
soil_reduced_thick soil_ness	thickness of soil horizons with a reduced matrix that covered 95% or more of the soil aggregates (in cm).
soil_histic_depth	depth of the occurrence of histic features in the soil profile (in cm).
soil_histic_thickness	thickness of histic features in the soil profile (in cm).
soil_organic_thickness	thickness of the organo-mineral horizon in the soil profile (in cm).
soil_moist_0_50	soil moisture at a depth of 0-50 cm. Expressed in ordinal categories: ‘1_dry’, ‘2_fresh’, ‘3_moist’, ‘4_damp’ or ‘5_ waterlogged’.
soil_moist_50_120	soil moisture at a depth of 50-120 cm. Expressed in ordinal categories: ‘1_dry’, ‘2_fresh’, ‘3_moist’, ‘4_damp’ or ‘5_ waterlogged’.

## Experimental Design, Materials and Methods

Wetland maps were used to select 377 sites throughout mainland France and Corsica from a variety of hydrogeomorphic systems, including ‘riverine or estuarine fringe’, ‘depressional’, ‘slope’, ‘lacustrine fringe’ and ‘flat’ [4]. Sites with damaged or artificially created wetlands were excluded. At each site, three transects were pre-located along an expected topographic moisture gradient using GIS based on 1:50,000 scale national geological maps (BRGM), 1:25,000 scale national hydrological and topographical maps (SCAN 25® from IGN), aerial photographs (BD ORTHO® from IGN) and, when available, regional wetland inventories. The distance between transects ranged from 100 to 200 m. Along each transect, the distance between successive plots ranged from 30–100 m. The distances were increased or decreased depending on practical access conditions in the field. Along each transect, at least one plot was sampled inside the wetland, and one plot was sampled outside the wetland. The number of plots per transect depended on the difficulty in identifying wetland boundaries in the field.

The geographical coordinates of each plot were recorded using GPS. Using a handbook, each plot was assigned to a natural habitat according to the European Nature Information System (EUNIS) [5] based on observed plant species and local environmental conditions [3]. Since redoximorphic features are indicators of wetland occurrence [6], the soil was sampled with a hand auger to determine the depth of occurrence and thickness of redox features (i.e. mottles that represented 5% or more of a given horizon), horizons with reduced matrix (grayish, bluish or greenish colors that covered 95% or more of a given horizon) and histic features (peaty materials). The sampling depth depended on the depth of occurrence of redoximorphic features: less than 50 cm deep if no redoximorphic features were observed, down to 80 cm if redoximorphic features occurred above 25 cm and down to 120 cm if redoximorphic features occurred only below 25 cm. The sampling depth depended also on possible obstacles existed (e.g. stones). The thickness of the organo-mineral horizon was measured, and soil moisture at depths of 0–50 cm and 50–120 cm was categorized in five ordinal categories.

## Ethics Statements

The authors declare that there are no ethical issues with the data presented.

## CRediT authorship contribution statement

**Guillaume Gayet:** Conceptualization, Methodology, Validation, Investigation, Writing – original draft, Writing – review & editing. **François Botcazou:** Investigation. **Jean-Manuel Gibeault-Rousseau:** Investigation. **Laurence Hubert-Moy:** Conceptualization, Methodology, Writing – original draft, Writing – review & editing. **Sébastien Rapinel:** Conceptualization, Methodology, Writing – original draft. **Blandine Lemercier:** Conceptualization, Methodology, Validation, Investigation, Writing – review & editing.

## Declaration of Competing Interest

The authors declare that they have no known competing financial interests or personal relationships that influenced the work described in this article.

The authors declare the following financial interests/personal relationships which may be considered as potential competing interests.

## Data Availability

Dataset of punctual observations along soil moisture gradients in France (Original data) (Dryad).
